# Robust modeling of additive and nonadditive variation with intuitive inclusion of expert knowledge

**DOI:** 10.1093/genetics/iyab002

**Published:** 2021-01-23

**Authors:** Ingeborg Gullikstad Hem, Maria Lie Selle, Gregor Gorjanc, Geir-Arne Fuglstad, Andrea Riebler

**Affiliations:** 1 Department of Mathematical Sciences, Norwegian University of Science and Technology, 7034 Trondheim, Norway; 2 The Roslin Institute and Royal (Dick) School of Veterinary Studies, University of Edinburgh, Easter Bush, Midlothian, EH25 9RG, Edinburgh

**Keywords:** Bayesian analysis, expert knowledge, genomic selection, hierarchical variance decomposition, nonadditive genetic variation

## Abstract

We propose a novel Bayesian approach that robustifies genomic modeling by leveraging expert knowledge (EK) through prior distributions. The central component is the hierarchical decomposition of phenotypic variation into additive and nonadditive genetic variation, which leads to an intuitive model parameterization that can be visualized as a tree. The edges of the tree represent ratios of variances, for example broad-sense heritability, which are quantities for which EK is natural to exist. Penalized complexity priors are defined for all edges of the tree in a bottom-up procedure that respects the model structure and incorporates EK through all levels. We investigate models with different sources of variation and compare the performance of different priors implementing varying amounts of EK in the context of plant breeding. A simulation study shows that the proposed priors implementing EK improve the robustness of genomic modeling and the selection of the genetically best individuals in a breeding program. We observe this improvement in both variety selection on genetic values and parent selection on additive values; the variety selection benefited the most. In a real case study, EK increases phenotype prediction accuracy for cases in which the standard maximum likelihood approach did not find optimal estimates for the variance components. Finally, we discuss the importance of EK priors for genomic modeling and breeding, and point to future research areas of easy-to-use and parsimonious priors in genomic modeling.

## Introduction

Plant breeding programs are improving productivity of a range of crops and with this addressing the global and rising hunger problem that impacts 820 million people across the world (FAO *et al.* 2019). One of the most important food sources in the world is wheat (Shewry and Hey 2015), however, recent improvements in wheat yield are smaller than the projected requirements (Ray *et al.* 2013) and might become more variable or even decrease due to climate change (Asseng *et al.* 2015). This trend is in stark contrast to the United Nation’s Sustainable Development Goals that aim to end hunger and malnutrition by 2030 (UN General Assembly 2015). Breeding has contributed significantly to the improvement of wheat yields in the past (*e.g.*, Mackay *et al.* 2011; Rife *et al.* 2019), and the recent adoption of genomic selection could enable further significant improvements (Gaynor *et al.* 2017; Belamkar *et al.* 2018; Sweeney *et al.* 2019).

Breeding programs generate and evaluate new genotypes with the aim to improve key characteristics such as plant height, disease resistance, and yield. Nowadays, a key component in breeding is genomic modeling, where we aim to reduce environmental noise in phenotypic observations and associate the remaining variation with variation in individual genomes. We use these associations to estimate genetic values for phenotyped or even nonphenotyped individuals and with this identify the genetically best individuals (Meuwissen *et al.* 2001). Improving this process involves improving the methods for disentangling genetic variation from environmental variation.

Genetic variation can be decomposed into additive and nonadditive components (Fisher 1918; Falconer and Mackay 1996; Lynch and Walsh 1998; Mäki-Tanila and Hill 2014). Additive variation is defined as variation of additive values, which are sums of allele substitution effects over the unobserved genotypes of causal loci. Statistically, the allele substitution effects are coefficients of multiple linear regression of phenotypic values on causal genotypes coded in an additive manner. Nonadditive variation is defined as the remaining genetic variation not captured by the additive values. It is commonly partitioned into dominance and epistasis values. Dominance values capture deviations from additive values at individual loci. Epistasis values capture deviations from additive and dominance values at combinations of loci. Statistically, dominance and epistasis values capture deviations due to allele interactions at individual loci and combinations of loci, respectively. Modeling interactions between two loci at a time give additive-by-additive, additive-by-dominance, and dominance-by-dominance epistasis. Modeling interactions between a larger number of loci increase the number of interactions.

Estimates of genetic values and their additive and nonadditive components have different applications in breeding (Acquaah 2007). Breeders use estimates of additive values to identify parents of the next generation, because additive values indicate the expected change in mean genetic value in the next generation under the assumption that allele frequencies will not change. Breeders use estimates of genetic values to identify individuals for commercial production, because genetic values indicate the expected phenotypic value. Estimates of genetic values are particularly valuable in plant breeding where individual genotypes can be effectively cloned. While genomic modeling currently focuses on additive values (Meuwissen *et al.* 2001; Varona *et al.* 2018), the literature on modeling nonadditive variation is growing (Oakey *et al.* 2006; Wittenburg *et al.* 2011; Muñoz *et al.* 2014; Bouvet *et al.* 2016; Martini *et al.* 2017; Vitezica *et al.* 2017; Varona *et al.* 2018; de Almeida Filho *et al.* 2019; Santantonio *et al.* 2019; Tolhurst *et al.* 2019; Martini *et al.* 2020). Notably, modeling nonadditive variation has been shown to improve the estimation of additive values in certain cases (Varona *et al.* 2018).

However, modeling nonadditive variation is challenging because it is difficult to separate nonadditive variation from additive and environmental variation even when large datasets are available (*e.g.*, Misztal 1997; Zhu *et al.* 2015; de los Campos *et al.* 2019). Furthermore, pervasive linkage and linkage disequilibrium are challenging the decomposition of genetic variance into its components (Gianola *et al.* 2013; Morota and Gianola 2014; Morota *et al.* 2014). This suggests that genomic modeling needs *robust* methods that do not estimate spurious nonadditive values and whose inference is *stable* for all sample sizes.

One way to handle partially confounded sources of variation is to take advantage of expert knowledge (EK) on their absolute or relative sizes. Information about the relative magnitude of the sources of phenotypic variation has been collated since the seminal work of Fisher (1918). The magnitude of genetic variation for a range of traits is well known (*e.g.*, Houle, 1992; Falconer and Mackay, 1996; Lynch and Walsh, 1998). Data and theory indicate that the majority of genetic variation is captured by additive values (Hill *et al.* 2008; Mäki-Tanila and Hill 2014), while the magnitude of variation in dominance and epistasis values varies considerably due to a range of factors. For example, there is no dominance variation between inbred individuals by definition. Furthermore, model specification has a strong effect on the resulting estimates (*e.g.*, Huang and Mackay 2016; Martini *et al.* 2017; Vitezica *et al.* 2017; Martini *et al.* 2020). With common model specifications, additive values capture most of the genetic variation because they capture the main effects (in the statistical sense), while dominance and epistasis values capture interaction deviations from the main effects (Hill *et al.* 2008; Mäki-Tanila and Hill 2014; Hill and Mäki-Tanila 2015; Huang and Mackay 2016). This EK does not need to come directly from the literature, it can also be formed based on internal estimates for a similar population in the past, or be a combination of both.

In a Bayesian setting, we can take advantage of such EK through prior distributions; see (Gianola and Fernando 1986; Sorensen and Gianola 2007) for an introduction to Bayesian methods in animal breeding and quantitative genetics, respectively. Prior distributions reflect beliefs and uncertainties about unknown quantities of a model and should be elicited from an expert in the field of interest (O’Hagan *et al.* 2006; Farrow 2013). Intuitively, prior distributions allow EK to act as additional observations, and make the current analysis more robust by borrowing strength from past analyses. Priors can improve weak identifiability of the sources of variation by guiding inference toward EK when the information in the sample is low. However, quantification of the effective number of samples added by a prior is only available in specific situations (Morita *et al.* 2008).

**Figure 1:**
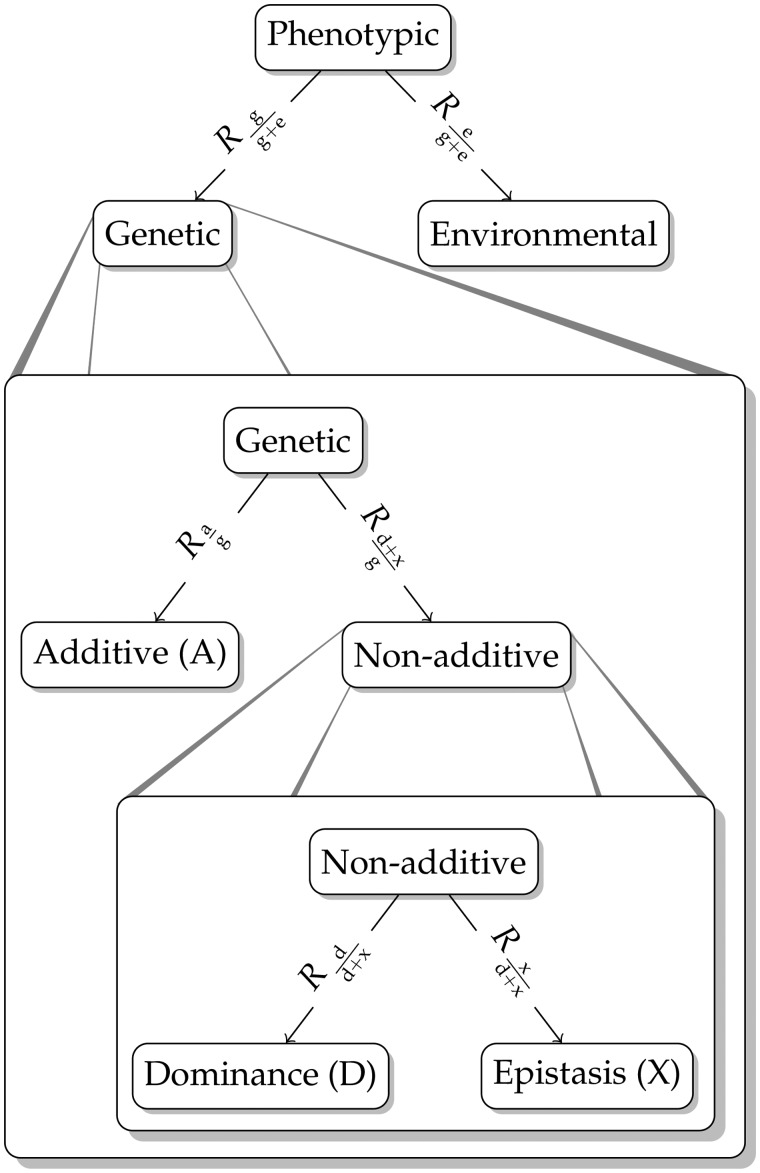
Tree structure visualizing the three possible model formulations A, AD, and ADX. Edge labels illustrate where EK applies, namely on the relative magnitude of the genetic and environmental variation and the relative magnitude of the additive, dominance, and epistasis variation.

**Figure 2:**
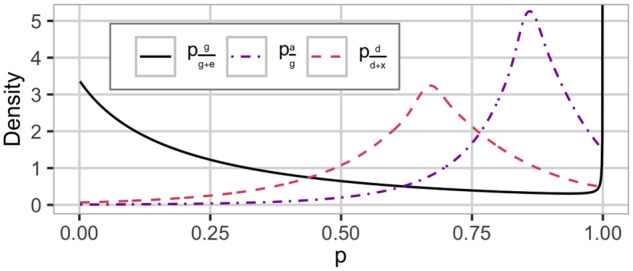
The HD prior used in the ADX-tree*^a^. setting with the proportion of genetic to phenotypic variance $p_{\frac{g}{g+e}}$, additive to genetic variance $p_{\frac{a}{g}}$, and dominance to nonadditive variance $p_{\frac{d}{d+x}}$. We use $R_{\frac{g}{g+e}}=0.25,\;R_{\frac{a}{g}}=0.85$, and $R_{\frac{d}{d+x}}=0.67$. This is a dataset specific prior. ^a^Additive and nonadditive model with model-wise EK prior.

We propose an easy-to-use, intuitive, and robust Bayesian approach that builds on two recent innovations in Bayesian statistics: (1) the hierarchical decomposition (HD) prior framework (Fuglstad *et al.* 2020) to provide a hierarchical description of the decomposition of phenotypic variation into different types of variation, and (2) the penalized complexity (PC) prior framework (Simpson *et al.* 2017) to facilitate robust genomic modeling. The key ideas of the approach are that (1) visualization eases model specification and communication about the model (see Figure 1), (2) HD of variation makes it easy to incorporate EK on *e.g.* heritability, (3) leveraging EK provides robust methods, and (4) comparison of posterior distributions and prior distributions reveal the amount of information the data provided on the decomposition of variation.

The aim of this paper is to demonstrate the new approach and to evaluate the potential impact of using the approach along with EK in plant breeding. We first describe the genomic model and how to incorporate the EK in this model. To test the proposed approach, we first use a simulated wheat breeding program and evaluate inference stability, estimation of genetic values, and variance components with different priors and with the standard maximum likelihood (ML) estimation. We also investigate the impact of dataset size. Then we apply the approach to a publicly available wheat yield dataset with 1739 individuals from 11 different trials in 6 locations in Germany with varying amounts of observed phenotypes from Gowda *et al.* (2014) and Zhao *et al.* (2015). We use cross-validation to assess the accuracy of phenotype prediction when using the proposed priors in the model. A description of the simulated and real wheat breeding case studies, model fitting, and analysis follows. Our key focus is to demonstrate how an analyst can take advantage of EK from literature or domain experts to enable robust genomic modeling of additive and nonadditive variation. This focus involves specifying and visualizing the EK in an intuitive way. We then present the results and discuss the relevance of our work.

## Materials and methods

### Genomic model

We model observed phenotypic values of *n* individuals $y=(y_{1},\ldots ,y_{n})$ with the aim to estimate their genetic values and their additive and nonadditive components. To this end, we use the genomic information about the individuals contained in the observed single nucleotide polymorphism (SNP) matrix **Z**, where row *i* contains SNP marker genotypes for individual *i* coded additively with 0, 1, 2. We let $Z_{a}$ be the column-centered **Z** where we have removed markers with low minor allele frequency, and let $Z_{d}$ be the column-centered matrix obtained from **Z** after setting heterozygote genotypes to 1 and homozygote genotypes to 0.

We model the phenotypic observation *y_i_* of individual *i* as:

(1) $$y_{i}=\mu +g_{i}+e_{i},\;\;i=1,\ldots ,n,$$

where *μ* is an intercept, *g_i_* is the genetic value, and *e_i_* the environmental residual for individual *i*. We model the environmental residual as an independently and identically distributed Gaussian random variable, $e=(e_{1},\ldots ,e_{n})∼N(0,\sigma_{e}^{2}I_{n})$, where $\sigma_{e}^{2}$ is the environmental variance and $I_{n}$ is the *n *×* n* identity matrix. The intercept is typically well-identified from the data, and we specify the nearly translation-invariant prior μ∼N(0,1000).

We consider the simple additive model with *g_i_* = *a_i_* (*Model A*), and nonadditive extension with dominance $g_{i}=a_{i}+d_{i}$ (*Model AD*), and epistasis $g_{i}=a_{i}+d_{i}+x_{i}$ (*Model ADX*). Here, $a=(a_{1},\ldots ,a_{n}),\;d=(d_{1},\ldots ,d_{n})$ and $x=(x_{1},\ldots ,x_{n})$, respectively, denote vectors of the additive, the dominance and the epistasis values for the individuals. Figure 1 shows the model structure for all three models, where every added component extends the model tree by one level. Moving from the root downwards, Model A is defined by the first split. Here only the additive value represents the genetic value. Model AD is defined by the first two splits, and as such has one level more. The genetic value splits into additive and nonadditive values, where only the dominance value represents the nonadditive value. Model ADX is defined by the complete tree and the nonadditive value consists of both dominance and epistasis values.

We model the genetic values as $a∼N(0,\sigma_{a}^{2}A)$, $d∼N(0,\sigma_{d}^{2}D)$, and $x∼N(0,\sigma_{x}^{2}X)$, where $\sigma_{a}^{2},\;\sigma_{d}^{2}$ and $\sigma_{x}^{2}$ are the additive, dominance, and epistasis variances, respectively. We specify the covariance matrices as $A=Z_{a}Z_{a}^{T}/S_{a}$, $D=Z_{d}Z_{d}^{T}/S_{d}$, and $X=A⊙A/S_{x}$ (we consider only additive-by-additive epistatis), where ⊙ is the Hadamard product (Henderson 1985; Horn 1990; Gianola and de los Campos 2008; Vitezica *et al.* 2017). To incorporate our EK in a unified way, we scale the covariance matrices with $S_{a},\;S_{d}$, and $S_{x}$ according to Sørbye and Rue (2018). The idea of such scaling is not new, see Legarra (2016), Vitezica *et al.* (2017), and Fuglstad *et al.* (2020) for details. Finally, the phenotypic variance is $\sigma_{P}^{2}=\sigma_{g}^{2}+\sigma_{e}^{2}=\sigma_{a}^{2}+\sigma_{d}^{2}+\sigma_{x}^{2}+\sigma_{e}^{2}$.

### EK about variance components

As highlighted in the introduction, there is prior information about the relative magnitude of the genetic and environmental variation and the relative magnitude of the additive, dominance, and epistasis variation that can guide the construction of prior distributions. We specify this EK in a hierarchical manner:

#### EK-pheno:

informs on the split of phenotypic variation into genetic and environmental variation. The proportion of genetic to phenotypic variation is denoted as $R_{\frac{g}{g+e}}=\frac{\sigma_{g}^{2}}{\sigma_{p}^{2}}=h_{g}^{2}$, where $h_{g}^{2}$ is the broad-sense heritability.

#### EK-genetic:

informs on the split of genetic variation into additive and nonadditive variation. The proportion of additive to genetic variation is denoted as $R_{\frac{a}{g}}=\frac{\sigma_{a}^{2}}{\sigma_{g}^{2}}=\frac{h_{a}^{2}}{h_{g}^{2}}$, where $h_{a}^{2}$ is the narrow-sense heritability.

#### EK-nonadd:

informs on the split of non-additive variation into dominance and epistasis variation. The proportion of dominance to nonadditive variation is denoted as $R_{\frac{d}{d+x}}=\frac{\sigma_{d}^{2}}{\sigma_{g}^{2}-\sigma_{a}^{2}}=\frac{h_{d}^{2}}{h_{g}^{2}-h_{a}^{2}}$, where $h_{d}^{2}$ is the proportion of dominance to phenotypic variation.

Figure 1 illustrates where the respective EK in the form of relative magnitudes $R_{*}$ applies. Of note, for Model A only EK-pheno is used, and EK-genetic is one ($R_{\frac{a}{g}}=1$) as nonadditive effects are not considered in this model. Similarly, for Model AD only EK-pheno and EK-genetic are used as EK-nonadd is one ($R_{\frac{d}{d+x}}=1$).

Values for the relative magnitudes $R_{*}$ will vary between study systems and traits in line with the EK. In this study, our knowledge is based on the cited literature in the introduction and practical experience with the analysis of a range of datasets. We follow the fact that many complex traits in agriculture are under sizeable environmental effect and that additive effects capture most genetic variation by standard quantitative model construction. With this in mind, we assume EK-pheno to be $R_{\frac{g}{g+e}}=0.25$, EK-genetic to be $R_{\frac{a}{g}}=0.85$, and EK-nonadd to be $R_{\frac{d}{d+x}}=0.67$. This implies $R_{\frac{d}{g}}=0.15\cdot 0.67\approx 0.10$ and $R_{\frac{x}{g}}=0.15\cdot 0.33\approx 0.05$. We emphasize that we use this information to construct prior distributions, *i.e.*, these relative magnitudes are only taken as a guide and not as the exact magnitude of variance components. Fuglstad *et al.* (2020) show that the prior for the first partition, the broad-sense heritability $h_{g}^{2}$, is not very influential.

We present two approaches for constructing a prior based on EK-pheno, EK-genetic, and EK-nonadd: (1) a component-wise (comp) prior, that is placed independently on each variance parameter; and (2) a tree-based (tree) model-wise prior that is defined jointly for all variance parameters. Both approaches are motivated by the concept of PC priors (Simpson *et al.* 2017).

### PC priors

A PC prior for a parameter *θ* is typically controlled by: (1) a preferred parameter value *θ*_0_ which is intuitive or leads to a simpler model; and (2) an idea on how strongly we believe in *θ*_0_. The PC prior shrinks toward *θ*_0_, unless the data indicate otherwise. This is achieved by constructing the prior based on a set of well-defined principles, for details, we refer to Simpson *et al.* (2017). PC priors can be applied to a standard deviation or variance, a proportion of variances, or other parameters such as correlations (Guo *et al.* 2017).

The PC prior for a standard deviation (*σ*) of a random effect will shrink the standard deviation toward zero, that is, toward a simpler model without the corresponding random effect (assuming the prior mean of the effect is zero). This prior is denoted as $\sigma ∼{\text{PC}}_{0}(\sqrt{V},\alpha )$ and results in an exponential distribution with rate parameter $-\ln(\alpha )/\sqrt{V}$. The subscript 0 in ${\text{PC}}_{0}(\cdot )$ indicates that the prior shrinks toward *σ *= 0. To define the prior the analyst has to specify an upper value $\sqrt{V}$ and a tail probability *α* such that the upper-tail probability $P(\sigma >\sqrt{V})=\alpha$. Here, we use α=0.25 so the prior distribution is weakly informative toward $\sqrt{V}$, but shrinks to zero unless the data inform otherwise.

For a variance proportion p∈[0,1], we denote the PC prior as $p∼{\text{PC}}_{0}(R)$. The numerical value R∈[0,1] encodes the available EK about the proportion and is set as the median of the prior, *i.e.* P(p>R)=0.5. The subscript 0 indicates that the prior shrinks toward *P *=* *0. Shrinkage toward the median is achieved by the PC prior $p∼{\text{PC}}_{M}(R)$, where *R* has the same interpretation as for ${\text{PC}}_{0}(R)$. For ${\text{PC}}_{M}(R)$, we need to specify how concentrated the distribution is on logit-scale in the interval [logit(R)−1,logit(R)+1] around the median [see Fuglstad *et al.* (2020) for details]. We allocated 75% probability to this interval.

The PC prior for a variance proportion depends on the structure of the two random components that are involved through their covariance matrices. We omit this in the notation for simplicity, and to emphasize that we chose to make the marginal priors on the proportions independent of each other. As the PC prior on proportions depends on the covariance matrix structure, it is application specific, and the priors do not correspond to common families of distributions such as the exponential or normal distributions [see Riebler *et al.* (2016) and Fuglstad *et al.* (2020) for more details].

### Component-wise prior

In the component-wise setting, we use a PC prior for each standard deviation parameter $\sigma_{*}$. The PC prior on $\sigma_{*}$ requires an upper value $\sqrt{V_{*}}$, so in addition to the relative magnitudes specified through EK-pheno, EK-genetic, and EK-nonadd we need information on the magnitude of the phenotypic variance to set up the component-wise priors. For this purpose, we could calculate the empirical phenotypic variance $V_{P}$ from a separate dataset, which is a trial study or a study believed to exhibit similar phenotypic variance as the study at hand. From this, we can define the upper values for the individual PC priors. For example, to formulate priors for Model A, we use EK-pheno to find $\sigma_{a}∼{\text{PC}}_{0}\left(\sqrt{h_{g}^{2}V_{P}},0.25\right)$ and $\sigma_{e}∼{\text{PC}}_{0}\left(\sqrt{(1-h_{g}^{2})V_{P}},0.25\right)$. For Model AD, we need EK-pheno and EK-genetic to formulate the priors, and for Model ADX, the most complex model, we take into account all available EK.

We follow the tree-structure shown in Figure 1 downwards to define the upper values, and multiply the relative magnitudes on the edges leading to the respective leaf nodes. For Model ADX, this leads us to:

- $\sigma_{e}∼{\text{PC}}_{0}\left(\sqrt{(1-h_{g}^{2})V_{P}},0.25\right)$,
- $\sigma_{a}∼{\text{PC}}_{0}\left(\sqrt{h_{a}^{2}V_{P}},0.25\right)$,
- $\sigma_{d}∼{\text{PC}}_{0}\left(\sqrt{h_{d}^{2}V_{P}},0.25\right)$, and
- $\sigma_{x}∼{\text{PC}}_{0}\left(\sqrt{(h_{g}^{2}-h_{a}^{2}-h_{d}^{2})V_{P}},0.25\right)$.

Combining the available EK procedure with the three different genomic models gave us settings, we denote as A-comp*, AD-comp*, and ADX-comp*. We have contrasted these settings with a *default* component-wise PC prior proposed by Simpson *et al.* (2017) with $\sqrt{V}=0.968$ and α=0.01 on all variance parameters, which gave us settings denoted as A-comp, AD-comp, and ADX-comp. This default prior is a prior without any EK. Preliminary analyses showed that the inferences for AD-comp, AD-comp*, ADX-comp, and ADX-comp* are not stable, *i.e.* the methods are not robust in the sense that they did not avoid estimating spurious nonadditive effects, and we do not present results from these settings. The priors for A-comp* and A-comp are plotted in Supplementary Figure S1 in File S1 in the Supplementary materials using $h_{g}^{2}=0.25$ and $V_{P}=1$. If $V_{P}$ takes another value, we simply rescale the *x*- and *y*-axes; the shape of the prior stays the same. In the simulated case study, we will use $V_{P}=1.86$. The priors are equal on all standard deviations for A-comp, AD-comp, and ADX-comp. The priors for AD-comp* and ADX-comp* can be seen in Supplementary Figures S2 and S3 in File S1. See Supplementary Note S1 in File S1 for a detailed description of the component-wise prior and posterior distributions for Model A and Model AD.

### Tree-based model-wise prior

In the model-wise setting, we shift the focus in Figure 1 from the leaf nodes to the splits. In other words, a shift from the component-wise perspective of variances associated with different sources of variation to a model-wise perspective of how these variances arise as a HD of the phenotypic variance. This provides a complementary way to construct priors where EK-pheno, EK-genetic, and EK-nonadd are directly incorporated at the appropriate levels in the tree structure. We achieve this by applying the HD prior framework of Fuglstad *et al.* (2020). We focus the presentation on the essential ideas for understanding and successfully applying the priors, and provide the comprehensive and mathematical description in Supplementary Note S1 in File S1. We emphasize that in the following $p_{*}$ denotes an actual variance proportion that we will infer (along with variances), while $R_{*}$ denotes EK for this proportion.

We first assign a marginal prior for the decomposition of variances in the lowest split, and then move step-wise up the tree assigning a prior to the decomposition of variance in each split conditional on the splits below it. The bottom-up process ends with the assignment of a prior to the root split, and the result is a joint prior for the decomposition of phenotypic variance into the different sources of variance. In the final step, we assign a prior for phenotypic variance $\sigma_{P}^{2}$ that is conditionally independent of the prior on the decomposition of the phenotypic variance.

We follow Fuglstad *et al.* (2020) and simplify the prior at each split by conditioning on EK from the lower splits. For example, the prior for $p_{\frac{a}{g}}$ is constructed under the assumption that $p_{\frac{d}{d+x}}=R_{\frac{d}{d+x}}$; that is, $\pi (p_{\frac{a}{g}}|p_{\frac{d}{d+x}})$ is replaced with $\pi (p_{\frac{a}{g}}|p_{\frac{d}{d+x}}=R_{\frac{d}{d+x}})$. Note that even though we construct the prior from the bottom and up, the arrows in the tree indicate how the phenotypic variance is distributed in the model from the top down. This means that the amount of, for example, dominance variance $\sigma_{d}^{2}$ depends on the variance partitions further up, since $\sigma_{d}^{2}=\sigma_{P}^{2}p_{\frac{g}{g+e}}(1-p_{\frac{a}{g}})p_{\frac{d}{d+x}}$ following the tree structure (Figure 1).

In this study, we assumed that at the lower levels the model shrinks toward the expert knowledge EK-nonadd and EK-genetic by using ${\text{PC}}_{M}(\cdot )$ priors. Furthermore, at the top level, we use a ${\text{PC}}_{0}(\cdot )$ prior to shrink toward the environmental effect unless the data indicate otherwise to reduce overfitting. Note that we could have chosen different assumptions. To obtain a prior fulfilling our assumptions, we follow four steps:

1. we use a ${\text{PC}}_{M}(\cdot )$ prior for the proportion of dominance to nonadditive variance with median $R_{\frac{d}{d+x}}=\frac{h_{d}^{2}}{h_{g}^{2}-h_{a}^{2}}$ (EK-nonadd),
2. we use a ${\text{PC}}_{M}(\cdot )$ prior for the proportion of additive to genetic variance with median $R_{\frac{a}{g}}=\frac{h_{a}^{2}}{h_{g}^{2}}$ (EK-genetic),
3. we use a ${\text{PC}}_{0}(\cdot )$ prior for the proportion of genetic to phenotypic variance with median $R_{\frac{g}{g+e}}=h_{g}^{2}$ (EK-pheno), and
4. we achieve scale-independence through the noninformative and scale-invariant Jeffreys’ prior for the phenotypic variance $\sigma_{P}^{2}∼1/\sigma_{P}^{2}$.

This construction gives the joint prior $\pi (\sigma_{P}^{2},p_{\frac{g}{g+e}},p_{\frac{a}{g}},p_{\frac{d}{d+x}})=\pi (\sigma_{P}^{2})\pi (p_{\frac{g}{g+e}})\pi (p_{\frac{a}{g}})\pi (p_{\frac{d}{d+x}})$ for Model ADX, where the conditioning on EK from lower splits is omitted to simplify notation. We denote this setting as ADX-tree* and show this prior in Figure 2 for $R_{\frac{g}{g+e}}=0.25,\;R_{\frac{a}{g}}=0.85$ and $R_{\frac{d}{d+x}}=0.67$. Note that the model-wise priors with EK are dependent on the covariance matrices of the modeled effects and are therefore dataset specific (Fuglstad *et al.* 2020), and the plots of these priors thus pertain to one specific dataset. The spike at *P *=* *1 for $p_{\frac{g}{g+e}}$ in Figure 2 is an artifact of the parameterization chosen for visualization and does not induce overfitting; see Fuglstad *et al.* (2020) for details. See Supplementary Note S1 in File S1 for a detailed description of the model-wise prior and posterior distributions for Model A and Model AD.

We explored the influence of alternative EK. In addition to the previously stated values for EK-pheno, EK-genetic, and EK-nonadd we also tested $R_{\frac{g}{g+e}}=0.25$, $R_{\frac{a}{g}}=0.05$, and $R_{\frac{d}{d+x}}\approx 0.11$ (so $R_{\frac{d}{g}}\approx 0.95\cdot 0.11\approx 0.10$ and $R_{\frac{x}{g}}\approx 0.95\cdot 0.89\approx 0.85$). The constructions follow the description above but with these relative magnitudes instead. We denote this setting as ADX-tree-opp*, as it expresses almost opposite or “wrong” beliefs compared to ADX-tree* setting, and show the prior in Supplementary Figure S4 in File S1 in the Supplementay materials.

**Figure 3:**
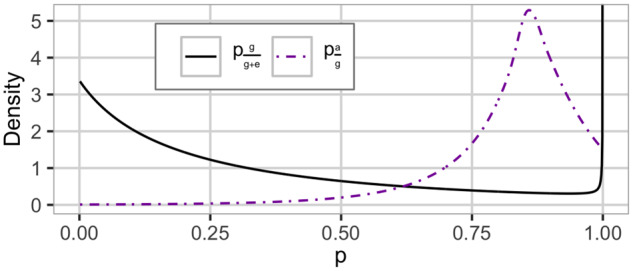
The HD prior used in the AD-tree*^a^ setting with the proportion of genetic to phenotypic variance $p_{\frac{g}{g+e}}$ and additive to genetic variance $p_{\frac{a}{g}}$. We use $R_{\frac{g}{g+e}}=0.25$ and $R_{\frac{a}{g}}=0.85$. This is a dataset specific prior. ^a^Additive and dominance model with model-wise EK prior.

For Model AD, the nonadditive effect only consists of dominance, and the variance is attributed to the different effects as visualized by the top and middle split in Figure 1. We construct a prior using EK-pheno and EK-genetic with $R_{\frac{g}{g+e}}=0.25$ and $R_{\frac{a}{g}}=0.85$ and denote this setting AD-tree*. The prior is shown in Figure 3.

**Figure 4:**
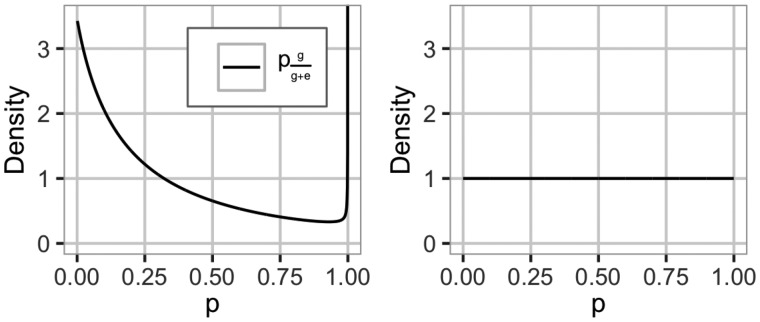
The prior for the proportion of genetic to phenotypic variance $p_{\frac{g}{g+e}}$ for the A-tree*^a^ (left) and A-tree^b^ (right) settings. We use $R_{\frac{g}{g+e}}=0.25$. A-tree^b^ is a dataset specific prior. ^a^Additive model with model-wise EK prior. ^b^Additive model with model-wise default prior. (A) Genetic value (variety selection). (A) Additive value (parent selection).

For Model A, the genetic variance is not decomposed to different sources and the distribution of the phenotypic variance can be visualized using the top split in Figure 1. We use EK-pheno with $R_{\frac{g}{g+e}}=0.25$ to construct a prior for the proportion of genetic to phenotypic variance and denote this setting as A-tree*. We show this prior in Figure 4.

We compared the model-wise prior with EK to a default prior with no EK by constructing an HD prior using the exchangeable Dirichlet prior on the proportion of phenotypic variance attributed to each of the sources of variance following Fuglstad *et al.* (2020). For Model A, we use a uniform prior, which is a special case of the symmetric Dirichlet(m) prior with *m *=* *2, on the proportion of genetic to phenotypic variance $p_{\frac{g}{g+e}}$ and denote this setting as A-tree (see Figure 4). For Models AD and ADX, we use Dirichlet(3) and Dirichlet(4) priors on the proportions, respectively, and denote these settings AD-tree and ADX-tree. These priors do not induce shrinkage toward any of the effects, and assume that each model effect contributes equally to the phenotypic variance, which due to the lack of EK did not lead to stable inference for Models AD and ADX. We do not show results from these settings. The tree structure and prior for AD-tree and ADX-tree can be seen in Supplementary Figures S5 and S6 in File S1, respectively. We summarize the model-wise priors that will be used in the following in Table 1.

**Table 1:** Summary of the model-wise (tree-based) prior distributions on proportions^a^**^,^^*b*^ and total phenotypic variance**

Additive (Model A, *g_i_* = *a_i_*)		**Additive and dominance (Model AD,** $g_{i}=a_{i}+d_{i}$ **)**	**Additive and nonadditive (Model ADX,** $g_{i}=a_{i}+d_{i}+x_{i}$ **)**

*Default*	*Expert*	*Expert*	*Expert*
A-tree: $\begin{array}{cc} p_{\frac{g}{g+e}} & ∼\text{Dirichlet}\left(2\right)\\ \sigma_{P}^{2} & ∼\text{Jeffrey}s^{\prime } \end{array}$	A-tree*: $\begin{array}{cc} p_{\frac{g}{g+e}} & ∼{\text{PC}}_{0}\left(R_{\frac{g}{g+e}}\right)\\ \sigma_{P}^{2} & ∼\text{Jeffrey}s^{\prime } \end{array}$	AD-tree*: $\begin{array}{cc} p_{\frac{g}{g+e}} & ∼{\text{PC}}_{0}\left(R_{\frac{g}{g+e}}\right)\\ p_{\frac{a}{g}} & ∼{\text{PC}}_{M}\left(R_{\frac{a}{g}}\right)\\ \sigma_{P}^{2} & ∼\text{Jeffrey}s^{\prime } \end{array}$	ADX-tree*: $\begin{array}{cc} p_{\frac{g}{g+e}} & ∼{\text{PC}}_{0}\left(R_{\frac{g}{g+e}}\right)\\ p_{\frac{a}{g}} & ∼{\text{PC}}_{M}\left(R_{\frac{a}{g}}\right)\\ p_{\frac{d}{d+x}} & ∼{\text{PC}}_{M}\left(R_{\frac{d}{d+x}}\right)\\ \sigma_{P}^{2} & ∼\text{Jeffrey}s^{\prime }\; \end{array}$

a $p∼{\text{PC}}_{0}(R)$ describes a PC prior for a variance proportion that has median equal to *R* and a preference for the variance proportion being equal to 0.

b $p∼{\text{PC}}_{M}(R)$ describes a PC prior for a variance proportion with median *R* and a preference for the variance proportion being equal to the median *R*, with 75% probability in [logit(R)−1,logit(R)+1] around the median on logit-scale.

### Simulated case study

We applied the described genomic model (1) with the above mentioned priors to a simulated case study that mimics a wheat breeding program to investigate the properties of the different settings. We simulated the breeding program using the R package AlphaSimR (Faux *et al.* 2016; Gaynor 2019) and closely followed the breeding program descriptions of Gaynor *et al.* (2017) (see their Figure 3) and Selle *et al.* (2019).

Specifically, we simulated a wheat-like genome with 21 chromosomes, selected at random, 1000 SNP markers and 1000 causal loci from each chromosome and used this genome to initiate a breeding program with breeding individuals. Every year, we have used 50 fully inbred parents to initiate a new breeding cycle with 100 random crosses. In each cross, we have generated 10 progenies and selfed them to generate 1000 F2 (second filial) individuals, which were selfed again to generate 10,000 F3 (third filial) individuals. We reduced the 10,000 F3 individuals in four successive selection stages (headrow, preliminary yield trial, advanced yield trial, and elite yield trial) with 10% selection intensity in each stage. In the headrow stage, we simulated a visual selection on a phenotype with the heritability of 0.03. For the preliminary, advanced and elite yield trials we, respectively, simulated selection on phenotype with heritability 0.25, 0.45, and 0.62. We used the 50 individuals with the highest phenotype values from the last three selection stages as parents for the next breeding cycle.

We ran the simulation for 30 years. At year 1, we set the following variances for the population of the 50 parents: additive variance of 1.0, dominance variance of 0.5, and epistasis variance of 0.1. We set the environmental variance to 6.0 for all stages and years. We ran the simulation for 20 years as a “burn-in” to obtain realistic breeding data under selection. We then ran the simulation for another 10 years with selection on phenotype. We removed the SNP markers with minor allele frequency below 5%. We did not use the models for selection.

### Real case study

We also applied the described genomic model (1) to the publicly available Central European wheat grain yield data from Gowda *et al.* (2014) and Zhao *et al.* (2015). In short, the data consist of 120 female and 15 male parent lines, which were crossed to create 1604 hybrids. The parents and hybrids were phenotyped for grain yield in 11 different trials in 6 locations in Germany. The number of observed phenotypes for the parents and hybrids vary between the trials, *i.e.*, some datasets have more observed phenotypes than others, ranging from 834 to 1739 (see Supplementary Table S1 in File S1 in the Supplementary materials). The parents and hybrids have genotype data for 17,372 high-quality SNP markers.

In the real case study, we analyzed the performance of the tree-based priors using EK (tree*) for the additive model (A), the additive and dominance model (AD), and the additive and nonadditive model (ADX). We used the same as in the simulation study: $R_{\frac{a}{g}}=0.85$ and $R_{\frac{d}{d+x}}=0.67$. We have, however, used a higher value in EK-pheno, $R_{\frac{g}{g+e}}=0.75$, in line with Reif *et al.* (2011)—later stage trials tend to have higher heritablity than early stage trials. Again, we emphasize that these values are only used to construct prior distributions and are not taken as literal proportions. The resulting priors can be seen in Supplementary Figure S7 in File S1.

### Implementation details

We fitted the models with a Bayesian approach through the R package RStan (Carpenter *et al.* 2017; Stan Development Team 2019). This package provides a sampling algorithm that uses the No-U-Turn sampler, a variant of Hamiltonian Monte Carlo, and only requires that the user specifies the joint posterior distribution up to proportionality, without having to write a sampling algorithm. See Supplementary Note S1 in File S1 for details. Sampling methods such as Markov Chain Monte Carlo and Hamiltonian Monte Carlo have guaranteed asymptotic accuracy as the number of drawn samples goes to infinity. However, in an applied context with finite computational resources, it is hard to assess this accuracy. Betancourt (2016) developed a diagnostic metric for the Hamiltonian Monte Carlo, called divergence, which indicates whether the sampler is able to transition through the posterior space effectively or not, where in the latter case the results might be biased (we show an example on this in the results).

We also fitted Models A, AD, and ADX with the ML approach using the low-storage BFGS (Broyden-Fletcher-Goldfarb-Shanno) algorithm through the R package nloptr (Nocedal 1980; Liu and Nocedal 1989; Johnson 2020). This approach does not use priors. We denote them as A-ML, AD-ML, and ADX-ML and use them as a baseline for comparison because ML is a common approach in the literature. Inference for ADX-ML was not robust, and we do not present results from this setting. At last, we compared the model results to the performance of selection based solely on phenotype where we treat the phenotype as a point estimate of the genetic value.

### Performance assessment

For the simulated case study, we ran the breeding program simulation 4000 times and fitted the model and prior settings in each of the last 10 years of simulation (40,000 model fits in total) at the third selection stage (advanced yield trial) in the program. Here we had 100 individuals each with five replicates and the goal was to select the 10 genetically best individuals for the fourth, last, stage. For each model fit, we evaluated: (1) robustness of method, (2) the accuracy of selecting the genetically best individuals, (3) the accuracy of estimating the different genetic values, and (4) the accuracy of estimating the variance parameters. We evaluated the fits against the true (simulated) values.

We measure how *robust* the method (model and inference approach) is, *i.e.*, to which degree it avoids estimating spurious nonadditive effects, in *stability of inference*. For the stability of inference of the Bayesian approach with Stan, we used the proportion of analyses that had stable inference (which we define as at least 99% samples where no divergent transitions were observed) for each model and prior setting. For the stability of inference of the ML approach we used the proportion of analyses where the optimizer algorithm converged.

For the accuracy of selecting the genetically best individuals, we ranked the best 10 individuals based on the estimated genetic value or estimated additive value, and counted how many were among the true genetically best 10 individuals based on the true genetic value or true additive value. We used the posterior mean of the effects as estimated values for ranking. Selection on the genetic value indicated selection of new varieties, while the selection on the additive value indicated selection of new parents.

For the accuracy of estimating the different genetic values (total genetic, additive, dominance, and epistasis values) we used Pearson correlation and continuous rank probability score (CRPS, Gneiting and Raftery 2007). With the correlation, we measured how well posterior means of genetic values correlated with true values (high value is desired). This metric works with point estimates and ignores uncertainty of inferred posterior distributions of each individual genetic value. The CRPS is a proper scoring rule and as such measures a combination of bias and sharpness of the posterior distribution compared to true values (low value is desired). Specifically, CRPS integrates squared difference between the cumulative estimated posterior distribution and the true value over the whole posterior distribution (Gneiting and Raftery 2007). See Selle *et al.* (2019) for a detailed explanation of CRPS used in a breeding context. In the case of phenotypic selection, we have a phenotype value for selection candidates, which is a point estimate of the genetic value, and its CRPS then reduces to the mean absolute error between the true genetic values and the phenotype.

The accuracy of the estimates of the variance parameters was assessed by dividing them by the true genetic variances for each of the 10 years from the simulated breeding program (a value close to 1 is desired). This is not done for phenotype selection.

To test the effect of dataset size on inference, we ran the breeding program an additional 1000 times and fitted the models to n=700,600,…,100 individuals in the preliminary stage (instead to 100 individuals in the advanced stage) at year 21. We used the settings with tree-based EK priors and the ML approach and investigated the performance of the methods for increasing number of observations by evaluating the robustness, and the accuracy of estimating the different genetic values and variance parameters.

We analyzed the real case study with the same models and tree-based EK priors and focused on the ability of predicting observed phenotypes in a cross-validation scheme. We performed fivefold cross-validations five times for each of the 11 trials independently. For each fold in each cross-validation, we predicted the held-out phenotypes (their posterior distribution involving intercept, genetic value, and environmental variation), and calculated the correlation between the point predictions and the observed phenotypes, and the CRPS using the phenotype posterior prediction distributions and the observed phenotypes available for each trial. We note that phenotype posterior predictions involve environmental variation, which does not affect point predictions and correlations, but affects the CRPS as the whole distribution of the prediction is involved in the calculations. We also looked at the posterior medians of the model variances. Of note, in contrast to the simulated case study, the genetic effects are unknown for real data, so that we cannot assess the estimation accuracy of the effects.

### Data and code availability

We provide code to simulate the data described in the simulated case study (Supplementary File S2). We also provide example code to fit the models presented in this paper together with an example dataset (Supplementary File S3). In the real case study, we used data from Gowda *et al.* (2014) (SNP genotypes) and Zhao *et al.* (2015) (phenotypes), and provide code for fitting the models in Supplementary File S4, including the folds used in the cross-validation. The Supplementary materials are available at figshare https://doi.org/10.6084/m9.figshare.12040716.

## Results

### Simulated case study

In the simulated case study, the model-wise priors and EK improved the inference stability of the nonadditive models and the selection of the genetically best individuals, but did not significantly improve the accuracy of estimating different genetic values for all individuals or for variance components.

#### Robustness and stability:

Table 2 shows the proportion of stable model fits by model and prior setting. The model-wise priors combined with EK improved the inference stability of the additive and dominant (AD) model and the nonadditive (ADX) model to the level of stability of the additive (A) model and phenotypic selection. Phenotypic selection does not depend on a model fit to a dataset and therefore had the highest method robustness by definition. This maximum level of robustness was matched by the simple additive model with the model-wise prior with and without using EK (A-tree* and A-tree) and with the standard ML approach (A-ML). This high model robustness was followed closely by fitting the more complicated nonadditive and additive and dominance models with model-wise prior and EK (ADX-tree* and AD-tree*). The Bayesian approach using component-wise priors with EK (A-comp*), the additive and dominance model with the ML approach (AD-ML), the component-wise priors without EK (A-comp), and the model-wise prior with wrong/opposite EK (ADX-tree-opp*) also resulted in satisfactory robustness, but then the proportion of model fits with stable inference started to decrease. The robustness of the additive and dominance model and the nonadditive model with default component-wise priors (AD-comp and ADX-comp) was improved by using the model-wise priors (AD-tree and ADX-tree), and even further by EK (AD-comp* and ADX-comp*), but neither they nor the nonadditive model fitted with ML (ADX-ML) had more than 80% stable model fits.

**Table 2:** Method robustness measured in stability of inference by model and prior setting

Setting (abbreviation)	Stability
Phenotype selection	1.00
Add. tree expert (A-tree*)	1.00
Add. tree default (A-tree)	0.99
Add. maximum likelihood (A-ML)	0.99
Non-add. tree expert (ADX-tree*)	0.98
Add. + dom. tree expert (AD-tree*)	0.97
Add. comp. expert (A-comp*)	0.94
Add. + dom. maximum likelihood (AD-ML)	0.88
Add. comp. default (A-comp)	0.86
Non-add. tree expert opposite (ADX-tree-opp*)	0.86
Non-add. comp. expert (ADX-comp*)	0.80
Non-add. maximum likelihood (ADX-ML)	0.79
Add. + dom. comp. expert (AD-comp*)	0.69
Add. + dom. tree default (AD-tree)	0.51
Non-add. tree default (ADX-tree)	0.23
Add. + dom. comp. default (AD-comp)	0.13
Non-add. comp. default (ADX-comp)	0.04

a As a proportion of analyses with less than 1% divergences for the Bayesian approach and as a proportion of analyses with convergence for the ML approach.

The reason for deteriorated robustness of some model and prior settings is that genetic (especially the nonadditive) and environmental effects can be partially confounded, which limits the exploration of the posterior when using the Bayesian approach or limits convergence of mode-seeking algorithms when using the ML approach. We show the partial confounding with images of the covariance matrices for additive, dominance, epistasis, and environmental sources of variation for one dataset in Supplementary Figure S8 in File S1, and scatterplots of the pairwise elements on and off the diagonal of the same matrices in Supplementary Figure S9. Supplementary Figure S10 shows joint posterior samples for the epistasis and environmental variance for model ADX with model-wise priors with and without EK (ADX-tree* and ADX-tree) for one dataset. Without a robust method (this includes both the model and inference approach), the posterior distribution becomes difficult to explore, and this is also supported by the divergence diagnostics (Table 2). The posterior of the ADX-tree setting is bimodal and the sampler has not been able to sample with convergence due to confounding.

We do not present results from the settings with 80% or less stable model fits (see Table 2) in the following. Note that Table 2 includes all model abbreviations used. For each setting, the breeding programs that did not result in stable inference were removed from the results.

#### Selecting best individuals:

**Figure 5:**
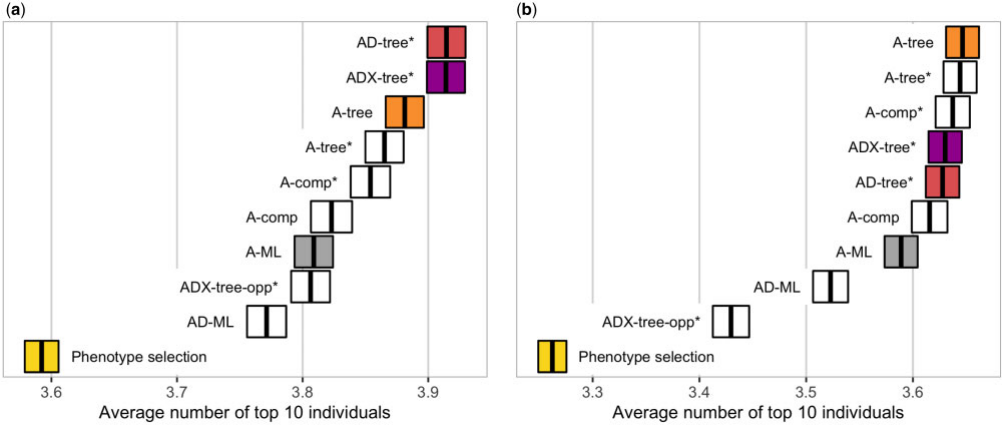
Accuracy of selecting individuals with the highest (A) genetic value (for variety selection) and (B) additive value (for parent selection) by model and prior setting—measured with the number of the top 10 true best individuals among the top 10 selected individuals (average ± two standard errors over replicates).

Figure 5 shows the accuracy of selecting individuals with the highest genetic value (variety selection, Figure 5A) and with the highest additive value (parent selection, Figure 5B). The model-wise priors exploiting EK improved the selection of the genetically best individuals significantly, and the model choice was important for different breeding aims. The settings with the additive and dominance model and the nonadditive model with model-wise EK (AD-tree* and ADX-tree*) performed significantly better in selection of new varieties than the others, which followed in order from A-tree, A-tree*, A-comp*, A-comp, A-ML, ADX-tree-opp*, and AD-ML (see Table 2 for abbreviations). The differences between the settings were small, but they all performed significantly better than sole phenotype selection, which is sensitive to environmental noise. For the selection of new parents, the simpler additive model performed the best, and the model-wise priors improved its performance (A-tree, A-tree*, and A-comp*). Wrong EK harmed the parent selection (ADX-tree-opp*), but it still outperformed sole phenotype selection.

#### Estimation:

We summarize the remaining results here, and include a detailed description of the results for the additive model with model-wise default prior (A-tree) and the ML approach (A-ML), the additive and dominance model and the nonadditive model with model-wise EK prior (AD-tree* and ADX-tree*), in addition to phenotype selection, in Supplementary Note S2, and provide the complete results for all settings in Supplementary Figures S11–S16 in File S1.

While using the model-wise priors and EK significantly improved the selection of the genetically best individuals compared to the ML approach, it did not significantly improve the accuracy of estimating different genetic values across all individuals (Supplementary Figures S11 and S12). There was a tendency for the Bayesian models to perform better than the models fitted with the ML approach, but the variation between replicates was larger than the variation between the settings. All models outperformed phenotype selection, where we treat the phenotype as a point estimate of the genetic value.

Supplementary Figure S13 shows that the variance component estimates varied considerably around the true values for all models and prior settings. The estimates from the Bayesian inference showed slightly larger biases and smaller variances than ML estimates. Estimates for epistasis variance were considerably more underestimated than for the dominance variance.

The inference stability did not increase with increasing number of observations for any of the models fitted with the ML approach. The Bayesian models with model-wise EK priors had the same high inference stability as in Table 2. The variation between replicates decreased for the variance estimates (Supplementary Figure S14) and the correlation and CRPS of the model effects improved for all models for increasing number of observations (Supplementary Figures S15 and S16). Seven hundred observations were not enough for the ML approach to obtain a bias in dominance and epistasis variance estimates as low as the Bayesian approach (Supplementary Figure S14), indicating that the need for good prior distributions is still there, but decreases with increasing number of observations.

### Real case study

**Figure 6:**
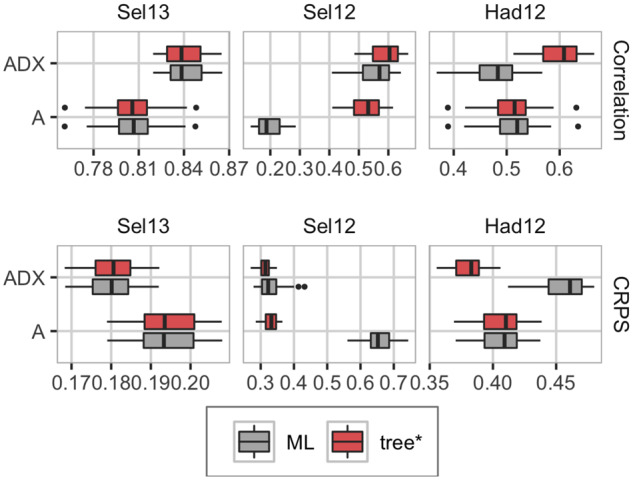
Phenotype prediction ability measured with correlation (top; high value desired), and CRPS (bottom; low value desired) from three of the trials in the real case study (boxplots show variation over the cross-validations and folds). Left: Sel13 (1739 observations), middle: Sel12 (834 observations), right: Had12 (1738 observations).

**Figure 7:**
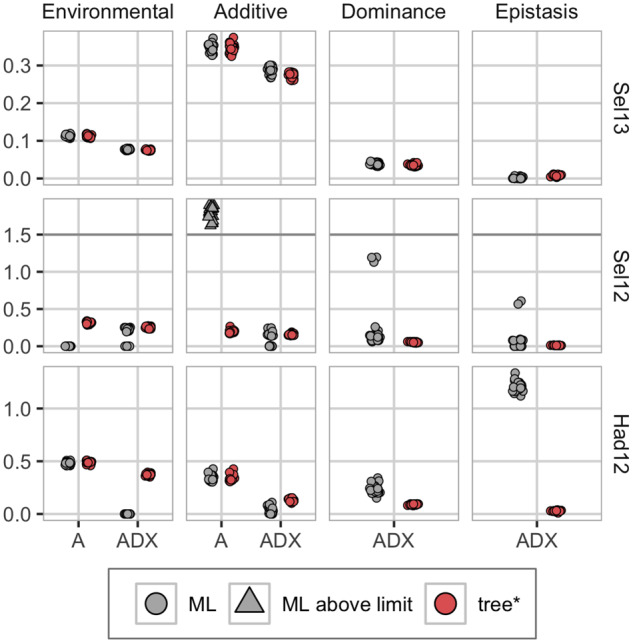
Posterior median variances from the real case study for three of the trials for the five fivefold cross-validations. Top: Sel13 (1739 observations), middle: Sel12 (834 observations), bottom: Had12 (1738 observations). For Sel12, the A-ML is overestimating the additive variance so badly (values over 400) that we have truncated the *y*-axes at 1.5 to highlight the other results.

The Bayesian approach with model-wise EK priors performed at least as good as or better than the ML approach. Figure 6 shows the predictive ability of phenotypes measured with correlation and CRPS from three trials in Seligenstadt (Sel13 and Sel12) and Hadmersleben (Had12) over the five fivefold cross-validations. These trials had phenotype observations for 1739 (Sel13), 834 (Sel12), and 1738 (Had12) parents and hybrids, and represent three different groups of trials: Sel13 represents the trials Ade13, Boh13, Hhof12, Hoh12, Hoh13, and Sel13 where few observations are missing and the Bayesian and ML approaches perform equally good. Sel12 represents the trials Boh12 and Sel12 where we have many missing observations and the ML approach is diverging. Had12 represents the trials Had12, Had13, and Hhof13 where few observations are missing but the ML approach leads to overfitting of the nonadditive effects. Inside each group, the results give similar conclusions, and we show results for only one trial in each group here. We include correlation and CRPS for all 11 trials in Supplementary Figures S17 and S18 in File S1. The ML approach was as good as the Bayesian approach in the Sel13 trial where all phenotypes were observed for the parents and hybrids, but in the Sel12 trial, which consists of only 834 out of 1739 observed phenotypes, the ML approach had worse predictive ability for the additive model (A), and slightly worse for the nonadditive model (ADX). In the Had12 trial with practically no unobserved phenotypes, the ML approach is outperformed by the Bayesian approach for the nonadditive model due to overfitting through overestimation of the epistasis variance (see Figure 7). The results from the additive and dominance (AD) model did not differ from the results from the additive and nonadditive model, and we to not discuss them here, but include the results from AD-tree* and AD-ML in Supplementary File S1 (Supplementary Figures S17–S19).

We explored reasons for the bad performance of A-ML in the Sel12 trial (representing trials with many missing observations). The ML optimizer returned a converge error message for two of the total 25 folds (we removed these model fits from all the results). However, the severe overestimation of the additive variance shown in Figure 7 indicates that the optimizer did not find the global maximum, but rather a local one. A closer investigation of the variance estimates showed that the optimizer got “stuck” at the lower boundary values (–20 for the environmental and –50 for the other variances on a logarithmic scale). We gave 0 as initial value for the intercept and log-variances for both the Bayesian and ML approach, however, the latter did not converge.

In Figure 7, we see that for Sel13 the approaches are in agreement on the variance estimates. With a dataset with many unobserved phenotypes (represented by Sel12), the additive model fitted with the ML approach (A-ML) estimated the environmental log-variance at –20, and in compensation severely overestimated the additive variance. The nonadditive model fitted with ML (ADX-ML) had the same underestimation of the environmental variance for some folds, but compensated with nonadditive effects. This indicates overfitting and means that predictions from such are based solely on genetic values, and no environmental effects, which gives misleading predictions. ADX-ML was also underestimating the environmental variance for the data from Had12, Had13, and Hhof13, and compensated this variance with the dominance and epistasis effects. We reran the ML optimizer with initial values set to posterior medians from the corresponding Bayesian models. In this case, the ML approach was not outperformed by the Bayesian approach (see Supplementary Figures S17 and S18 in File S1). The variance estimates for all environments can be seen in Supplementary Figure S19.

In Supplementary Figures S17 and S18, we see that the trend is the same across the trials; for datasets where we have observed most of the phenotypes for the parents and hybrids, the ML and Bayesian approaches are in general performing equally, and we gain predictive accuracy by including nonadditive effects, but as soon as there are many unobserved phenotypes, such as for Boh12 and Sel12 (see Supplementary Table S1 for information about all trials), the ML approach is deteriorating. For the Had12, Had13, and Hhof13 trials, which has few unobserved phenotypes but still has poor predictive abilities for the nonadditive model (ADX), the ML approach has problems with overfitting (see Supplementary Figure S19). The model underestimates the environmental variance and attributes this variation to the dominance and epistasis effects.

## Discussion

In this study, we have introduced new priors for robust genomic modeling of additive and nonadditive variation based on the PC prior (Simpson *et al.* 2017) and HD prior (Fuglstad *et al.* 2020) frameworks. In the simulated case study, the new priors enabled straightforward use of EK, which in turn improved the robustness of genomic modeling and the selection of the genetically best individuals in a wheat breeding program. However, it did not improve the overall accuracy of estimating genetic values for all individuals or for variance components. In the real case study, the new priors improved the prediction ability, especially for trials with fewer observations, and they reduced overfitting. These results highlight three points for discussion: (1) expert-knowledge priors for genomic modeling and prediction, (2) the importance of priors for breeding, and (3) limitations of our work.

### EK priors for genomic modeling and prediction

Genomic modeling is challenging due to inherent high-dimensionality and pervasive correlations between loci and therefore requires substantial amounts of information for robust estimation. Most genomes harbor millions of segregating loci that are highly or mildly correlated. While estimating additive effects at these loci is a challenging task in itself (*e.g.*, Visscher *et al.* 2017; Young 2019), estimating dominance and epistasis effects is an even greater challenge (*e.g.*, Misztal 1997; Zhu *et al.* 2015; de los Campos *et al.* 2019). One challenge in estimating the interactive dominance and epistasis effects is that they are correlated with the main additive effects and all these effects are further correlated across nearby loci (Mäki-Tanila and Hill 2014; Hill and Mäki-Tanila 2015; Vitezica *et al.* 2017). Information to estimate all these locus effects and corresponding individual values has to inherently come from the data, but could also come in a limited extent from the EK. There is a wealth of EK in genetics (*e.g.*, Houle 1992; Falconer and Mackay 1996; Lynch and Walsh 1998), however, this EK is seldom used because it is not clear how to use it in a credible and a consistent manner.

We showed how to use the EK about the magnitude of different sources of variation by leveraging two recently introduced prior frameworks (Simpson *et al.* 2017; Fuglstad *et al.* 2020). While the PC priors are parsimonious and intuitive, they require absolute prior statements when used in a component-wise approach, which are challenging to elicit for multiple effects. The HD framework imposes a tree structure according to a domain model, and the intuitive PC prior can be used in the HD prior framework to ensure robust modeling. This model-wise approach enables the use of *relative* prior statements, which are less challenging to elicit than the absolute prior statements, because we tend to have good knowledge of the broad-sense heritabililty for most traits and by the standard quantitative genetic model construction we know that additive effects capture majority of genetic variance (Hill *et al.* 2008; Mäki-Tanila and Hill 2014; Hill and Mäki-Tanila 2015; Huang and Mackay 2016). The presented priors therefore pave a way for a fruitful elicitation dialog between a geneticist and a statistician (Farrow 2013). In particular, the HD prior framework provides both a method for prior construction and a platform for communication among geneticists and statisticians. The model-wise EK prior must naturally be adapted to each model, as it depends on the model structure, but using the tree structures makes this adaption intuitive and should as such help with prior elicitation (O’Hagan *et al.* 2006; Farrow 2013). Furthermore, the graphical representation allows a description of a joint prior in a visual way with minimal statistical jargon (Figure 1).

An example of using such EK was the choices of a median for the broad-sense heritability of 0.25 in the simulated and 0.75 in the real case study. However, as Figure 2 and Supplementary Figure S7 show the priors do not differ tremendously. This shows that the prior proposed in this study is vague and not restricted by the value chosen for the median. Perhaps there is even scope for more concentrated priors, should such information be available.

The HD prior framework enabled us to use EK on relative additive and nonadditive variation. If nonadditive effects are to be added to the model, EK is necessary for the inference to be stable and the results reliable, and the simulation study shows that the EK must be added in such a way that the magnitude of the variances are not restricted by the prior, *i.e.*, the model-wise approach instead of the component-wise approach. In the simulated case study, the EK improved the stability of inference of the Bayesian approach over the ML approach and improved the selection of the genetically best individuals. This improvement was due the additional information that alleviated the strong confounding between the nonadditive (particularly epistasis) and environmental variation.

The HD prior framework is also useful when EK is only available on parts of the model. For example, an expert may not have a good intuition about the level of broad-sense heritability, say for a new trait, but will likely have a good intuition on how the genetic variance *relatively* decomposes into additive, dominance, and epistasis components, simply due to the model specification (Hill *et al.* 2008; Mäki-Tanila and Hill 2014; Hill and Mäki-Tanila 2015; Huang and Mackay 2016). In those cases, we can use weakly informative default priors on the parts of the model where EK is missing, and priors based on EK for the rest of the model. The component-wise specification of EK with the standard (Sorensen and Gianola 2007) or the PC (Simpson *et al.* 2017) priors is infeasible in this context, and does not admit a simple visualization of the implied assumptions on the decomposition of the phenotypic variance. Furthermore, the component-wise specification of EK is particularly challenging when phenotypic variance is unknown or when collected observations are influenced by a range of effects which can inflate sample phenotypic variance. The model-wise approach with the HD prior can address these situations.

There exists previous work on penalized estimation of genetic covariances (*e.g.*, Meyer *et al.* 2011; Meyer 2016, 2019) that also uses Bayesian principles and scale-free penalty functions to reduce variation of the estimates from small datasets and for large numbers of traits. Our proposed priors and EK reduced variation of estimates in the simulated case study. However, our estimates were biased, which is expected given the small sample size and that the Bayesian approach induced bias toward a lower variance (*e.g.*, Sorensen and Gianola 2007). It is worth noting that the ML estimates of genetic variance also were largely underestimated, which we believe is due to the small sample size and a large number of parameters to estimate. We see in Supplementary Note S2 (File S1) that the data inform about phenotypic variance and broad-sense heritability, but only weakly about the division of the additive and nonadditive, and dominance and epistasis. Furthermore, for some datasets, we could not obtain the ML estimates, while priors robustified the modeling by penalizing the genetic effects. The real case study also showed that using EK increases the inference robustness in datasets with a large amount of unobserved phenotypes, and reduces overfitting. We saw this improvement in both the Bayesian approach and the ML approach where we used the results from the Bayesian models as initial values for the optimization algorithm. However, the latter approach requires specific EK on the size of the variances, which in the same way as the component-wise EK priors, is difficult to elicit from experts in the field. We note, however, that genomic models are inherently misspecified by trying to estimate the effect of causal loci through correlated marker loci (Gianola *et al.* 2009; de los Campos *et al.* 2015). Also, linkage and linkage disequilibrium are challenging the decomposition of genetic variance into its components (Gianola *et al.* 2013; Morota *et al.* 2014; Morota and Gianola 2014). Indeed, our variance estimates were not very accurate in the simulated case study.

Future research could expand the HD prior framework to other settings. For example, to multiple traits or modeling genotype-by-environment interactions, which are notoriously noisy, and we aim to find parsimonious models (*e.g.*, Meyer 2016, 2019; Tolhurst *et al.* 2019). Also, expand to model macro- and micro-environmental effects (*e.g.*, Selle *et al.* 2019) and to model multiple layers of sparse, yet high-dimensional, “omic” data from modern biological experiments using network-like models (Damianou and Lawrence 2013).

### Importance of priors for breeding

Robust genomic modeling of nonadditive variation is important for breeding programs. There is substantial literature indicating sizeable nonadditive genetic variation (*e.g.*, Oakey *et al.* 2006; Muñoz *et al.* 2014; Bouvet *et al.* 2016; Varona *et al.* 2018; de Almeida Filho *et al.* 2019; Santantonio *et al.* 2019; Tolhurst *et al.* 2019), but robust modeling of this variation is often challenging. We have shown how to achieve this robust modeling with the proposed priors and EK. We evaluated this approach with a simulated wheat breeding program where we assessed the ability to select the genetically best individuals on their genetic value (variety selection) and additive value (parent selection). The results showed that the proposed priors and the EK improved variety and parent selection. We observed more improvement in the variety selection, which is expected because there is more variation in genetic values than its first-order approximation additive values. However, this additional nonadditive variation is hard to model due to a small signal from the data relative to environmental variation and confounding with the environmental variation. This confounding is expected. As pointed by one of the reviewers, we obtain the epistasis covariance matrix using the Hadamard product of the additive covariance matrix with itself, and such repeated Hadamard multiplication converges to an identity matrix, *i.e.*, to the covariance matrix of the environmental effect. Both the simulated and real case studies showed that including nonadditive effects in the model requires some sort of penalization to avoid overfitting environmental noise as nonadditive genetic effects. The proposed priors and the EK helped us to achieve this.

Importantly, all models improved upon sole phenotypic selection in the simulated case study, which shows the overall importance of genomic modeling While the differences between the different models and priors were small, the improved genomic modeling can contribute to the much needed improvements in plant breeding (Ray *et al.* 2013; Asseng *et al.* 2015). Also, even a small improvement in the variety selection has a huge impact on production, because varieties are used extensively (Acquaah 2007). In the terms of model complexity, the answer to whether to use the additive model, the additive and dominance model or the nonadditive model depended on the aim of the analysis. The latter models were the best in selecting the genetically best individuals on genetic value, whereas the additive model performed best in selecting the genetically best individuals on additive value. The reason for this is likely the small sample size and large number of parameters to estimate with the nonadditive model (Varona *et al.* 2018). In the real case study adding nonadditive effects to the model improved the phenotypic prediction accuracy beyond the additive model, and the EK helped us to avoid overfitting, which shows the advantage of the EK.

Of note is the observation that the proposed priors and the EK improved the selection of the genetically best individuals, but not the estimation of the different genetic values. We did not expect this difference. In principle, both of these metrics are important, but for breeding the ability to select the genetically best individuals is more important (de los Campos *et al.* 2013). A possible explanation for the difference between the two metrics is that the top individuals deviated more from the overall distribution and the overall metrics do not capture well the tail-behaviour.

The importance of the proposed priors and the EK will likely vary with the stage and size of a breeding program, and as the simulation study with increasing amount of observations and the real case study shows, the importance of priors increases with the decreasing amount of observations. Prior importance is known to decrease as the amount of data increases (Sorensen and Gianola 2007), but the required amount of data for accurate estimation of nonadditive effects is huge compared to the size of most breeding programs. Therefore, the proposed PC and HD priors could be helpful also in large breeding programs as they enforce shrinkage according to the EK unless the data indicate otherwise, reducing the risk of estimating spurious effects.

### Limitations of our work

The aim of this paper was to describe the use of the EK to improve genomic modeling, which we achieved through two recently introduced prior frameworks (Simpson *et al.* 2017; Fuglstad *et al.* 2020), and demonstrated their use in a simulated and a real case study of wheat breeding. There are multiple other possible uses of the proposed priors in genomic modeling and prediction. The simulated case study is small with only 100 individuals at the advanced yield trials of a wheat breeding program, and up to 700 individuals at the preliminary yield trials. A small number of individuals and a limited genetic variation at this stage made a good case study to test the importance of priors, and we show that using our approach can be beneficial beyond the standard genomic model. We have also chosen this stage for computationally simplicity and speed because we evaluate the robustness of estimation over many replicates. Studies with more individuals are a natural next step, but is beyond the scope of this paper due to computational reasons. Finally, we could have tested more prior options, in particular the shrinkage of the nonadditive values toward the additive values, *i.e.*, the ${\text{PC}}_{0}(\cdot )$ versus the ${\text{PC}}_{M}(\cdot )$ prior. More research is needed in the future to see how the EK can improve genetic modeling further.

Interesting areas for future research are also in other breeding domains with the recent rise in volumes of individual genotype and phenotype data, which provide power for estimating dominance and epistasis values (*e.g.*, Alves *et al.* 2020; Joshi *et al.* 2020). The ability to estimate the nonadditive values would be very beneficial in breeding programs that aim to exploit biotechnology (*e.g.*, Gottardo *et al.* 2019). Finally, an exciting area for estimating nonadditive individual values is in the area of personalized human medicine (de los Campos *et al.* 2010; Mackay and Moore 2014; Sackton and Hartl 2016; de los Campos *et al.* 2018; Begum 2019).

The proposed priors are novel and require further computational work to facilitate widespread use. The PC priors (Simpson *et al.* 2017) are increasingly used in the R-INLA software (Rue *et al.* 2009, 2017), while HD priors (Fuglstad *et al.* 2020) have been implemented with the general purpose Bayesian software Stan (Carpenter *et al.* 2017; Stan Development Team 2019). This implementation is technical and Stan is slow for genomic models, although there is active development to increase its computational performance (Margossian *et al.* 2020).

We are in the process of developing an R package that will offer an intuitive user interface to specify HD priors. The clear graphical representation of the priors along the model defined tree encourages increased transparency within the scientific community. It facilitates communication and discussion between statisticians and nonstatisticians in the process of the model design, prior specification but also model assessment. Existing EK is intuitively incorporated into PC prior distributions for the parameters where it applies to. The resulting model-wise prior can be fed directly into Stan or INLA, or can be precomputed for use in other Bayesian software. Thus, the new priors will be straightforward to apply for statisticians and nonstatisticians, robustify the analysis, and the use of INLA will speed up computations. Further work is needed to enable Bayesian treatment of large genomic models fitted to datasets with hundreds of thousands of individuals.

## Conclusion

In conclusion, we have presented how to use the EK on relative magnitude of genetic variation and its additive and nonadditive components in the context of a Bayesian approach with two novel prior frameworks. We believe that when modeling a phenomenon for which there exists a lot of knowledge, we should employ methods that are able to take advantage of this resource. We have demonstrated with a simulated and a real case study that such methods are important and helpful in the breeding context, and they might have potential also in other areas that use genomic modeling.

## Funding

I.G.H., G.-A.F., and A.R. were supported by project number 240873 from the Research Council of Norway. G.G. was supported by the BBSRC to the Roslin Institute (BBS/E/D/30002275) and the University of Edinburgh’s Data-Driven Innovation Chancellor’s fellowship.

## Conflicts of interest

None declared.

## References

[iyab002-B1] Acquaah G. 2007. Principles of Plant Genetics and Breeding. Oxford, UK: Blackwell.

[iyab002-B2] Alves K , Brito LF, Baes CF, Sargolzaei M, Robinson JAB, et al 2020. Estimation of additive and non-additive genetic effects for fertility and reproduction traits in North American Holstein cattle using genomic information. J Anim Breed Genet. 137:316–315. 31912573 10.1111/jbg.12466

[iyab002-B3] Asseng S , Ewert F, Martre P, Rötter RP, Lobell DB, et al 2015. Rising temperatures reduce global wheat production. Nat Clim Change. 5:143–147.

[iyab002-B4] Begum R. 2019. A decade of Genome Medicine: toward precision medicine. Genome Med. 11:13. 30819206 10.1186/s13073-019-0624-z PMC6394063

[iyab002-B5] Belamkar V , Guttieri MJ, Hussain W, Jarquín D, El-Basyoni I, et al 2018. Genomic selection in preliminary yield trials in a winter wheat breeding program. G3 (Bethesda). 8:2735–2747. 29945967 10.1534/g3.118.200415 PMC6071594

[iyab002-B6] Betancourt M. 2016. Diagnosing suboptimal cotangent disintegrations in Hamiltonian Monte Carlo. arXiv preprint arXiv:1604.00695 [stat.ME].

[iyab002-B7] Bouvet J-M , Makouanzi G, Cros D, Vigneron P. 2016. Modeling additive and non-additive effects in a hybrid population using genome-wide genotyping: prediction accuracy implications. Heredity. 116:146–157. 26328760 10.1038/hdy.2015.78 PMC4806881

[iyab002-B8] Carpenter B , Gelman A, Hoffman MD, Lee D, Goodrich B, et al 2017. Stan: a probabilistic programming language. J Stat Soft. 76:1–32. 10.18637/jss.v076.i01 PMC9788645 36568334

[iyab002-B9] Damianou A , Lawrence N. 2013. Proceedings of the Sixteenth International Conference on Artificial Intelligence and Statistics. PMLR. 31:207–215.

[iyab002-B10] de Almeida Filho JE , Guimarães JFR, Fonsceca e Silva F, Vilela de Resende MD, Muñoz P, et al 2019. Genomic prediction of additive and non-additive effects using genetic markers and pedigrees. G3 (Bethesda). 9:2739–2748. 31263059 10.1534/g3.119.201004 PMC6686920

[iyab002-B11] de los Campos G , Gianola D, Allison DB. 2010. Predicting genetic predisposition in humans: the promise of whole-genome markers. Nat Rev Genet. 11:880–886. 21045869 10.1038/nrg2898

[iyab002-B12] de los Campos G , Hickey JM, Pong-Wong R, Daetwyler HD, Calus MPL. 2013. Whole-genome regression and prediction methods applied to plant and animal breeding. Genetics. 193:327–345. 22745228 10.1534/genetics.112.143313 PMC3567727

[iyab002-B13] de los Campos G , Sorensen D, Gianola D. 2015. Genomic heritability: what is it? PLoS Genet. 11:e1005048. 25942577 10.1371/journal.pgen.1005048 PMC4420472

[iyab002-B14] de los Campos G , Sorensen DA, Toro MA. 2019. Imperfect linkage disequilibrium generates phantom epistasis (& perils of big data). G3 (Bethesda). 9:1429–1436. 30877081 10.1534/g3.119.400101 PMC6505142

[iyab002-B15] de los Campos G , Vazquez AI, Hsu S, Lello L. 2018. Complex-trait prediction in the era of big data. Trends Genet. 34:746–754. 30139641 10.1016/j.tig.2018.07.004 PMC6150788

[iyab002-B16] Falconer DS , Mackay TFC. 1996. Introduction to Quantitative Genetics, 4th ed. Harlow: Longman Group.

[iyab002-B17] FAO, IFAD, UNICEF, WFP, and WHO. 2019. The State of Food Security and Nutrition in the World 2019. Rome: FAO.

[iyab002-B18] Farrow M. 2013. Prior elicitation. In: Dubitzky W, Wolkenhauer O, Cho K-H, Yokota H, editors. Encyclopedia of Systems Biology. New York, NY: Springer New York. p. 1743.

[iyab002-B19] Faux A-M , Gorjanc G, Gaynor RC, Battagin M, Edwards SM, et al 2016. Alphasim: software for breeding program simulation. Plant Genome. 9:1–14. 10.3835/plantgenome2016.02.0013 27902803

[iyab002-B20] Fisher RA. 1918. The correlation between relatives on the supposition of Mendelian inheritance. Trans R Soc Edinb. 52:399–433.

[iyab002-B21] Fuglstad G-A , Hem IG, Knight A, Rue H, Riebler A. 2020. Intuitive joint priors for variance parameters. Bayesian Anal. 15:1109–1137.

[iyab002-B22] Gaynor C. 2019. AlphaSimR: Breeding Program Simulations. R package version 0.10.0. 10.1093/g3journal/jkaa017 PMC8022926 33704430

[iyab002-B23] Gaynor RC , Gorjanc G, Bentley AR, Ober ES, Howell P, et al 2017. A two-part strategy for using genomic selection to develop inbred lines. Crop Sci. 57:2372–2386.

[iyab002-B24] UN General Assembly. 2015. Transforming our world : the 2030 Agenda for Sustainable Development, 21 October 2015 A/RES/70/1.

[iyab002-B25] Gianola D , de los Campos G. 2008. Inferring genetic values for quantitative traits non-parametrically. Genet Res. 90:525–540. 10.1017/S0016672308009890 19123970

[iyab002-B26] Gianola D , de los Campos G, Hill WG, Manfredi E, Fernando R. 2009. Additive genetic variability and the Bayesian alphabet. Genetics. 183:347–363. 19620397 10.1534/genetics.109.103952 PMC2746159

[iyab002-B27] Gianola D , Fernando RL. 1986. Bayesian methods in animal breeding theory. J Anim Sci. 63:217–244.

[iyab002-B28] Gianola D , Hospital F, Verrier E. 2013. Contribution of an additive locus to genetic variance when inheritance is multi-factorial with implications on interpretation of GWAS. Theor Appl Genet. 126:1457–1472. 23508282 10.1007/s00122-013-2064-2

[iyab002-B29] Gneiting T , Raftery AE. 2007. Strictly proper scoring rules, prediction, and estimation. J Am Stat Assoc. 102:359–378.

[iyab002-B30] Gottardo P , Gorjanc G, Battagin M, Gaynor RC, Jenko J, et al 2019. A strategy to exploit surrogate sire technology in livestock breeding programs. G3 (Bethesda). 9:203–215. 30563834 10.1534/g3.118.200890 PMC6325890

[iyab002-B31] Gowda M , Zhao Y, Würschum T, Longin CF, Miedaner T, et al 2014. Relatedness severely impacts accuracy of marker-assisted selection for disease resistance in hybrid wheat. Heredity. 112:552–561. 24346498 10.1038/hdy.2013.139 PMC3998782

[iyab002-B32] Guo J , Riebler A, Rue H. 2017. Bayesian bivariate meta-analysis of diagnostic test studies with interpretable priors. Statist Med. 36:3039–3058. 10.1002/sim.7313 28474394

[iyab002-B33] Henderson CR. 1985. Best linear unbiased prediction of nonadditive genetic merits in noninbred populations. J Anim Sci. 60:111–117.

[iyab002-B34] Hill W , Mäki-Tanila A. 2015. Expected influence of linkage disequilibrium on genetic variance caused by dominance and epistasis on quantitative traits. J Anim Breed Genet. 132:176–186. 25823842 10.1111/jbg.12140

[iyab002-B35] Hill WG , Goddard ME, Visscher PM. 2008. Data and theory point to mainly additive genetic variance for complex traits. PLoS Genet. 4:e1000008. 18454194 10.1371/journal.pgen.1000008 PMC2265475

[iyab002-B36] Horn RA. 1990. The Hadamard Product. Proc Symp Appl Math. 40:87–169.

[iyab002-B37] Houle D. 1992. Comparing evolvability and variability of quantitative traits. Genetics. 130:195–204. 1732160 10.1093/genetics/130.1.195 PMC1204793

[iyab002-B38] Huang W , Mackay TFC. 2016. The genetic architecture of quantitative traits cannot be inferred from variance component analysis. PLoS Genet. 12:e1006421. 27812106 10.1371/journal.pgen.1006421 PMC5094750

[iyab002-B39] Johnson SG. 2020. The NLopt nonlinear-optimization package. (Accessed: 2020 July 17). http://ab-initio.mit.edu/nlopt.

[iyab002-B40] Joshi R , Meuwissen TH, Woolliams JA, Gjøen HM. 2020. Genomic dissection of maternal, additive and non-additive genetic effects for growth and carcass traits in Nile tilapia. Genet Sel Evol. 52:1. 31941436 10.1186/s12711-019-0522-2 PMC6964056

[iyab002-B41] Legarra A. 2016. Comparing estimates of genetic variance across different relationship models. Theor Popul Biol. 107:26–30. 26341159 10.1016/j.tpb.2015.08.005

[iyab002-B42] Liu DC , Nocedal J. 1989. On the limited memory BFGS method for large scale optimization. Math Program. 45:503–528.

[iyab002-B43] Lynch M , Walsh B. 1998. Genetics and Analysis of Quantitative Traits. MA: Sinauer Sunderland.

[iyab002-B44] Mackay I , Horwell A, Garner J, White J, McKee J, et al 2011. Reanalyses of the historical series of UK variety trials to quantify the contributions of genetic and environmental factors to trends and variability in yield over time. Theor Appl Genet. 122:225–238. 20835813 10.1007/s00122-010-1438-y

[iyab002-B45] Mackay TF , Moore JH. 2014. Why epistasis is important for tackling complex human disease genetics. Genome Med. 6:125. 25031624 10.1186/gm561 PMC4062066

[iyab002-B46] Mäki-Tanila A , Hill WG. 2014. Influence of Gene Interaction on Complex Trait Variation with Multilocus Models. Genetics. 198:355–367. 24990992 10.1534/genetics.114.165282 PMC4174947

[iyab002-B47] Margossian CC , Vehtari A, Simpson D, Agrawal R. 2020. Hamiltonian Monte Carlo using an adjoint-differentiated Laplace approximation. arXiv preprint arXiv:2004.12550.

[iyab002-B48] Martini JW , Gao N, Cardoso DF, Wimmer V, Erbe M, et al 2017. Genomic prediction with epistasis models: on the marker-coding-dependent performance of the extended GBLUP and properties of the categorical epistasis model (CE). BMC Bioinformatics. 18:3. 28049412 10.1186/s12859-016-1439-1 PMC5209948

[iyab002-B49] Martini JW , Toledo FH, Crossa J. 2020. On the approximation of interaction effect models by Hadamard powers of the additive genomic relationship. Theor Popul Biol. 132:16–23. 31991144 10.1016/j.tpb.2020.01.004

[iyab002-B50] Meuwissen THE , Hayes BJ, Goddard ME. 2001. Prediction of total genetic value using genome-wide dense marker maps. Genetics. 157:1819–1829. 11290733 10.1093/genetics/157.4.1819 PMC1461589

[iyab002-B51] Meyer K. 2016. Simple penalties on maximum-likelihood estimates of genetic parameters to reduce sampling variation. Genetics. 203:1885–1900. 27317681 10.1534/genetics.115.186114 PMC4981284

[iyab002-B52] Meyer K. 2019. “ Bending” and beyond: Better estimates of quantitative genetic parameters? J Anim Breed Genet. 136:243–251. 31247680 10.1111/jbg.12386

[iyab002-B53] Meyer K , Kirkpatrick M, Gianola D, et al 2011. Penalized maximum likelihood estimates of genetic covariance matrices with shrinkage towards phenotypic dispersion. Proc Ass Advan Anim Breed Genet. 19:87–90.

[iyab002-B54] Misztal I. 1997. Estimation of variance components with large-scale dominance models. J Dairy Sci. 80:965–974.

[iyab002-B55] Morita S , Thall PF, Müller P. 2008. Determining the effective sample size of a parametric prior. Biometrics. 64:595–602. 17764481 10.1111/j.1541-0420.2007.00888.x PMC3081791

[iyab002-B56] Morota G , Boddhireddy P, Vukasinovic N, Gianola D, DeNise S. 2014. Kernel-based variance component estimation and whole-genome prediction of pre-corrected phenotypes and progeny tests for dairy cow health traits. Front Genet. 5:56. 24715901 10.3389/fgene.2014.00056 PMC3970026

[iyab002-B57] Morota G , Gianola D. 2014. Kernel-based whole-genome prediction of complex traits: a review. Front Genet. 5:363. 25360145 10.3389/fgene.2014.00363 PMC4199321

[iyab002-B58] Muñoz PR , Resende MFR, Gezan SA, Resende MDV, de los Campos G, et al 2014. Unraveling additive from nonadditive effects using genomic relationship matrices. Genetics. 198:1759–1768. 25324160 10.1534/genetics.114.171322 PMC4256785

[iyab002-B59] Nocedal J. 1980. Updating quasi-Newton matrices with limited storage. Math Comp. 35:773–782.

[iyab002-B60] Oakey H , Verbyla A, Pitchford W, Cullis B, Kuchel H. 2006. Joint modeling of additive and non-additive genetic line effects in single field trials. Theor Appl Genet. 113:809–819. 16896718 10.1007/s00122-006-0333-z

[iyab002-B61] O’Hagan A , Buck CE, Daneshkhah A, Eiser JR, Garthwaite PH, et al 2006. Uncertain Judgements: Eliciting Experts’ Probabilities. West Sussex, UK: John Wiley & Sons.

[iyab002-B62] Ray DK , Mueller ND, West PC, Foley JA. 2013. Yield trends are insufficient to double global crop production by 2050. PLoS One. 8:e66428. 23840465 10.1371/journal.pone.0066428 PMC3686737

[iyab002-B63] Reif JC , Maurer HP, Korzun V, Ebmeyer E, Miedaner T, et al 2011. Mapping QTLs with main and epistatic effects underlying grain yield and heading time in soft winter wheat. Theor Appl Genet. 123:283–292. 21476040 10.1007/s00122-011-1583-y

[iyab002-B64] Riebler A , Sørbye SH, Simpson D, Rue H. 2016. An intuitive Bayesian spatial model for disease mapping that accounts for scaling. Stat Methods Med Res. 25:1145–1165. 27566770 10.1177/0962280216660421

[iyab002-B65] Rife TW , Graybosch RA, Poland JA. 2019. A field-based analysis of genetic improvement for grain yield in winter wheat cultivars developed in the US Central Plains from 1992 to 2014. Crop Sci. 59:905–910.

[iyab002-B66] Rue H , Martino S, Chopin N. 2009. Approximate Bayesian inference for latent Gaussian models by using integrated nested Laplace approximations. J R Stat Soc B. 71:319–392.

[iyab002-B67] Rue H , Riebler A, Sørbye SH, Illian JB, Simpson DP, et al 2017. Bayesian computing with INLA: a review. Annu Rev Stat Appl. 4:395–421.

[iyab002-B68] Sackton TB , Hartl DL. 2016. Genotypic context and epistasis in individuals and populations. Cell. 166:279–287. 27419868 10.1016/j.cell.2016.06.047 PMC4948997

[iyab002-B69] Santantonio N , Jannink J-L, Sorrells M. 2019. Prediction of subgenome additive and interaction effects in allohexaploid wheat. G3 (Bethesda). 9:685–698. 30455185 10.1534/g3.118.200613 PMC6404612

[iyab002-B70] Selle ML , Steinsland I, Hickey JM, Gorjanc G. 2019. Flexible modelling of spatial variation in agricultural field trials with the R package INLA. Theor Appl Genet. 132:3277–3293. 31535162 10.1007/s00122-019-03424-y PMC6820601

[iyab002-B71] Shewry PR , Hey SJ. 2015. The contribution of wheat to human diet and health. Food Energy Secur. 4:178–202. 27610232 10.1002/fes3.64 PMC4998136

[iyab002-B72] Simpson D , Rue H, Riebler A, Martins TG, Sørbye SH. 2017. Penalising model component complexity: a principled, practical approach to constructing priors. Statist Sci. 32:1–28.

[iyab002-B73] Sørbye SH , Rue H. 2018. Fractional Gaussian noise: prior specification and model comparison. Environmetrics. 29:e2457.

[iyab002-B74] Sorensen D , Gianola D. 2007. Likelihood, Bayesian, and MCMC Methods in Quantitative Genetics. New York: Springer Science & Business Media.

[iyab002-B75] Stan Development Team. 2019. RStan: the R interface to Stan. R package version 2.19.2. http://mc-stan.org/.

[iyab002-B76] Sweeney DW , Sun J, Taagen E, Sorrells ME. 2019. Genomic selection in wheat. In: Miedaner T, Korzun V, editors. Applications of Genetic and Genomic Research in Cereals. Woodhead Publishing Series in Food Science, Technology and Nutrition. Duxford, UK: Woodhead Publishing. p. 273–302.

[iyab002-B77] Tolhurst DJ , Mathews KL, Smith AB, Cullis BR. 2019. Genomic selection in multi-environment plant breeding trials using a factor analytic linear mixed model. J Anim Breed Genet. 136:279–300. 31247682 10.1111/jbg.12404

[iyab002-B78] Varona L , Legarra A, Toro MA, Vitezica ZG. 2018. Non-additive effects in genomic selection. Front Genet. 9:78. 29559995 10.3389/fgene.2018.00078 PMC5845743

[iyab002-B79] Visscher PM , Wray NR, Zhang Q, Sklar P, McCarthy MI, et al 2017. 10 years of GWAS discovery: biology, function, and translation. Am J Hum Genet. 101:5–22. 28686856 10.1016/j.ajhg.2017.06.005 PMC5501872

[iyab002-B80] Vitezica ZG , Legarra A, Toro MA, Varona L. 2017. Orthogonal estimates of variances for additive, dominance, and epistatic effects in populations. Genetics. 206:1297–1307. 28522540 10.1534/genetics.116.199406 PMC5500131

[iyab002-B81] Wittenburg D , Melzer N, Reinsch N. 2011. Including non-additive genetic effects in Bayesian methods for the prediction of genetic values based on genome-wide markers. BMC Genet. 12:74. 21867519 10.1186/1471-2156-12-74 PMC3748015

[iyab002-B82] Young AI. 2019. Solving the missing heritability problem. PLoS Genet. 15:e1008222. 31233496 10.1371/journal.pgen.1008222 PMC6611648

[iyab002-B83] Zhao Y , Li Z, Liu G, Jiang Y, Maurer HP, et al 2015. Genome-based establishment of a high-yielding heterotic pattern for hybrid wheat breeding. Proc Natl Acad Sci USA. 112:15624–15629. 26663911 10.1073/pnas.1514547112 PMC4697414

[iyab002-B84] Zhu Z , Bakshi A, Vinkhuyzen AA, Hemani G, Lee SH, et al 2015. Dominance genetic variation contributes little to the missing heritability for human complex traits. Am J Hum Genet. 96:377–385. 25683123 10.1016/j.ajhg.2015.01.001 PMC4375616

